# A 44K microarray dataset of the changing transcriptome in developing Atlantic salmon (*Salmo salar *L.)

**DOI:** 10.1186/1756-0500-4-88

**Published:** 2011-03-29

**Authors:** Stuart G Jantzen, Dan S Sanderson, Kris R von Schalburg, Motoshige Yasuike, Francesco Marass, Ben F Koop

**Affiliations:** 1 Center for Biomedical Research, University of Victoria, Victoria, British Columbia, V8W 3N5, Canada

## Abstract

**Background:**

Atlantic salmon (*Salmo salar *L.) is an environmentally and economically important organism and its gene content is reasonably well characterized. From a transcriptional standpoint, it is important to characterize the changes in gene expression over the course of unperturbed early development, from fertilization through to the parr stage.

**Findings:**

*S. salar *samples were taken at 17 time points from 2 to 89 days post fertilization. Total RNA was extracted and cRNA was synthesized and hybridized to a newly developed 44K oligo salmonid microarray platform. Quantified results were subjected to preliminary data analysis and submitted to NCBI's Gene Expression Omnibus (GEO). Data can be found under the GEO accession number GSE25938. http://www.ncbi.nlm.nih.gov/geo/query/acc.cgi?acc=GSE25938

**Conclusions:**

Throughout the entire period of development, several thousand genes were found to be differentially regulated. This work represents the trancriptional characterization of a very large geneset that will be extremely valuable in further examination of the transcriptional changes in Atlantic salmon during the first few months of development. The expression profiles can help to annotate salmon genes in addition to being used as references against any number of experimental variables to which developing salmonids might be subjected.

## Background

Atlantic salmon (*Salmo salar *L.) are an environmentally and economically important organism. The genome has been well studied and is currently being fully sequenced [1-4]. In addition, a number of microarrays have been developed for transcription studies of *S. salar *[2,5-7]. As a benefit of the extensive characterization of the transcriptome of *S. salar*, large scale studies of gene expression changes can be undertaken using these microarray platforms [8].

This study is the first to utilize a newly developed 44K oligo salmonid microarray design, one of the first salmonid oligo microarrays. This array comprises approximately 22,000 60-mer oligos that were conserved (95% similar) between rainbow trout (*Oncorhynchus mykiss*) and Atlantic salmon [6] plus 14,866 additional Atlantic salmon and 5,661 additional rainbow trout contig sequences. The result is a microarray that has a large transcript representation with very low redundancy. The array is composed of oligos based on roughly 80% *S. salar *and 20% *O. mykiss *contigs. 84% of all features are well annotated with fairly stringent hits (e-value cutoff: 1e-10) to public databases (December 17, 2009). The annotation files may be found at the cGRASP microarray page [9]. Efforts to annotate unknown contigs will continue.

Library construction, sequence analysis and contig assembly have been described previously [2]. The 14,866 additional *S. salar *oligos were all derived from selected contigs compiled in the local database of the authors (August 11, 2009) [10]. These were chosen first from the approximately 10,000 full length cDNAs in the database [11]. The remainder were selected from well annotated sequences and then from poorly annotated sequences with an open reading frame longer than 300 bp represented by two or more clones. 5,206 of the additional *O. mykiss *contig-derived oligos were selected from the set of well annotated sequences in the local database that did not have a clear homologous representative in *S. salar*. The remaining 455 were selected from annotated NCBI Nucleotide resources (July 21, 2009) [12] with priority given to immune system related sequences. Representative oligos from sequences identified by Gene Ontology (GO) [13] were included. After sequence selection, oligos were derived from the selected contigs by Agilent Technologies (Santa Clara, CA) and were 60 bp in length. The oligo selection process was biased in favor of 3' sequences. While the majority of oligos are unique to contigs (i.e. only one spot on the array can be mapped back to a given contig), approximately 27% of oligos, including the original 22,000, were derived from the same contig as at least one other oligo. Finally, in situ oligo synthesis and microarray manufacturing was performed by Agilent. Microarray slides are available through Agilent's eArray platform, with each slide containing four arrays [14].

Due to the very large number of unique features on this platform, a genome-wide exploration of expression levels in salmonids is expected to produce significant and detailed information on many molecular systems. For example, the genetic factors involved in the very early developmental stages of Atlantic salmon are not completely understood. It is therefore of interest to do a thorough examination of *S. salar *developmental stages from fertilization through to the parr stage, using a transcriptomics approach. Recently, another group has used a microarray platform to profile the changes in gene expression during smoltification in Atlantic salmon [15] when freshwater parr make the transition to saltwater smolt. The dataset presented here complements this earlier study.

The objective was to comprehensively monitor the salmonid transcriptome during controlled and unperturbed development. This work complements and facilitates recent efforts to sequence and annotate the Atlantic salmon genome. It further provides a resource that identifies expression levels of tens of thousands of genes during the course of development. These baseline expression patterns can be used as references in future experiments to examine physiological, reproductive, mutational, and environmental variables.

## Materials and methods

### Animals and sampling

Treatment of the fish used in this study was in compliance with the regulations of the University of Victoria Animal Care Committee. Eggs from Atlantic salmon (McConnell (Mowi)) were obtained in November, 2009 from Marine Harvest United Hatchery (Fanny Bay, BC, Canada). The eggs were fertilized by gently mixing the eggs and milt by hand and then washed with partial exchanges of water. Approximately 2,000 fertilized eggs were then transferred and placed in Heath trays (Marisource, Fife, WA) at the University of Victoria. The embryos and larvae were raised in fresh water at a temperature of 12°C and a flow rate of 200 liters/h.

**Figure 1:**
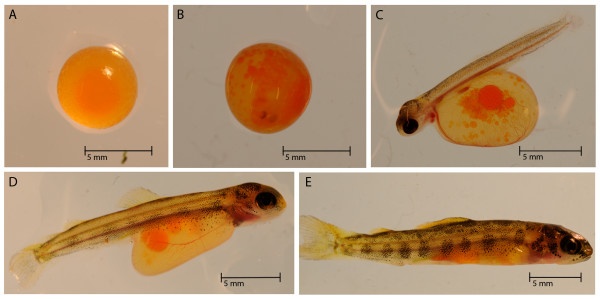
***S. salar *during development**. Photographs taken during development of Atlantic salmon at the University of Victoria. A) 5 B) 20 C) 41 D) 59 E) 77 dpf.

The day of fertilization was marked as 0 days post fertilization (dpf), and hatching and yolk sac absorption occurred between 38 to 40 dpf and 68 to 70 dpf, respectively. Whole embryos (n = 20) and larvae were collected every one to three days for several weeks. Alevin and fry were collected every sixth day for the remainder of the study (Figure 1). Unfertilized eggs were not included in the experimental design, which could be used to examine possible RNA effects from the oocyte. Samples were directly placed into dry ice and stored at -80°C until RNA extraction.

### RNA extraction

Total RNAs were extracted in TRIzol reagent (Invitrogen, Carlsbad, CA) by mixer-mill homogenization (Retsch, Newtown, PA) and spin-column purified using RNeasy Mini kits (Qiagen, Hilden, Germany). The RNA was extracted from three whole individual embryos, larvae, or alevins at 2 dpf and then every fifth day (5 to 35 dpf) or sixth day (35 to 89 dpf). Quantity and quality of RNA samples were then measured using UV absorbance (NanoDrop Technologies, Wilmington, DE) and quality was also checked by agarose gel electrophoresis.

### cRNA synthesis, labeling, and purification

Cy5 labeled experimental cRNA samples were generated using an Agilent Low Input Quick Amp (LIQA) kit, following the manufacturer's instructions. For each time point, 40 ng of total RNA from three individuals was used to generate first-strand cDNA. Agilent Spike-In B control RNA was included in each reaction. After the denaturation step (10 min at 65°C) and cRNA synthesis step (2 hr at 40°C), the reactions were incubated at 70°C for 15 minutes to inactivate the AffinityScript enzyme and subsequently stored at -80°C until further use. For the labeling reactions, thawed cRNA samples were each mixed with 16 μL of Transcription Master Mix cocktail containing Cy5 dye, and incubated at 40°C for two hours. Purification was performed using Qiagen RNeasy mini spin columns, eluting in 30 μL of RNase-free water. For the generation of the reference pool, equimolar amounts from the three individuals in each time point were pooled to give 120 ng of total RNA used in each first-strand reaction. Spike-In A control RNA was included in each reaction. After labeling with Cy3 and column purification as above, a common reference pool was created by including 2.8 μg of cRNA from each time point, except for 2 dpf, for which only 1.3 μg of material was ultimately available. Due to the small size and early developmental stage of samples from days 2, 5, and 10 dpf, limited RNA quantities necessitated additional extractions and subsequent synthesis and labeling reactions, however repeated procedures produced Cy5 labeled cRNA of the required quantity and quality.

### Microarray hybridization and scanning

Experimental samples of Cy5 labeled cRNA were quantified on a Nanodrop ND-1000. All samples were found to be of sufficient specific activity with a mean (± SD) of 18.22 ± 2.03 pmol Cy5/μg cRNA as per manufacturer's recommendation (Agilent) and an appropriate RNA absorbance ratio with a mean of 2.29 ± 0.06. Next, cRNA fragmentation mixtures were created following the LIQA kit instructions, using 825 ng of experimental sample and 825 ng of reference pool. These mixtures were incubated at 60°C for 30 minutes. After cooling on ice for one minute, hybridization mixtures were prepared by adding 2x GEx Hybridization Buffer HI-RPM and mixing well by pipetting. These reactions were loaded in random arrangements with respect to time point onto 44K oligo salmonid microarrays (Agilent-025055) using Agilent SureHyb Hybridization Chambers. Each of the 4 × 44K arrays on the microarray slides had 100 μL of hybridization reaction added. The hybridization reactions were allowed to occur for 17 hours at 65°C. Slide washes were performed as per the manufacturer's instructions, including an ozone-protection step using the Agilent Stabilization and Drying Solution. Slides were scanned as soon as possible on a ScanArray Express (PerkinElmer, Waltham, MA) scanner at 5 μm resolution using a PMT setting of 80 in both channels, a black threshold of 1800, and a full color threshold of 26.8. Slides were stored in a low ozone chamber (typically < 5 ppb) until scanned.

### Data processing

Since the temperature of the environment has a strong influence on the rate of development of Atlantic salmon [16], time points as dpf were converted to both degree days and to the relative age in terms of Tau-somite (τ_s_) as proposed by Gorodilov [17] (Table 1). This allowed for the determination of corresponding phenotypic stages independent of the temperature during development.

**Table 1:** *S. salar *developmental stages sampled

Days post fertilization (dpf)	Degree days (12°C * dpf)	**Relative age in τ**_**s**_	Subperiod
2	24	17	Blastulation

5	60	42	Gastrulation

10	120	83	Somitogenesis

15	180	125	Vascularization of yolksac

20	240	167	Vascularization of yolksac

25	300	208	Formation of caudal rays

30	360	250	Formation of caudal rays

35	420	292	Formation of caudal rays

41	492	342	Free embryo/Alevin

47	564	392	Free embryo/Alevin

53	636	442	Free embryo/Alevin

59	708	492	Alevins have left gravel/ Beginning of parr markings

65	780	542	Fry/Appearance of caudal parr marks

71	852	592	Fry/Yolk-sac completely absorbed

77	924	642	Parr

83	996	692	Parr

89	1068	742	Parr

*S. salar *samples were taken at 17 time points, which correspond to specific developmental stages depending on the environmental temperature. Developmental stage can be measured in degree days, as relative age in Tau-somite(τ_s_) [16], or described as a phenotypic subperiod as expected in the wild [16].

**Figure 2:**
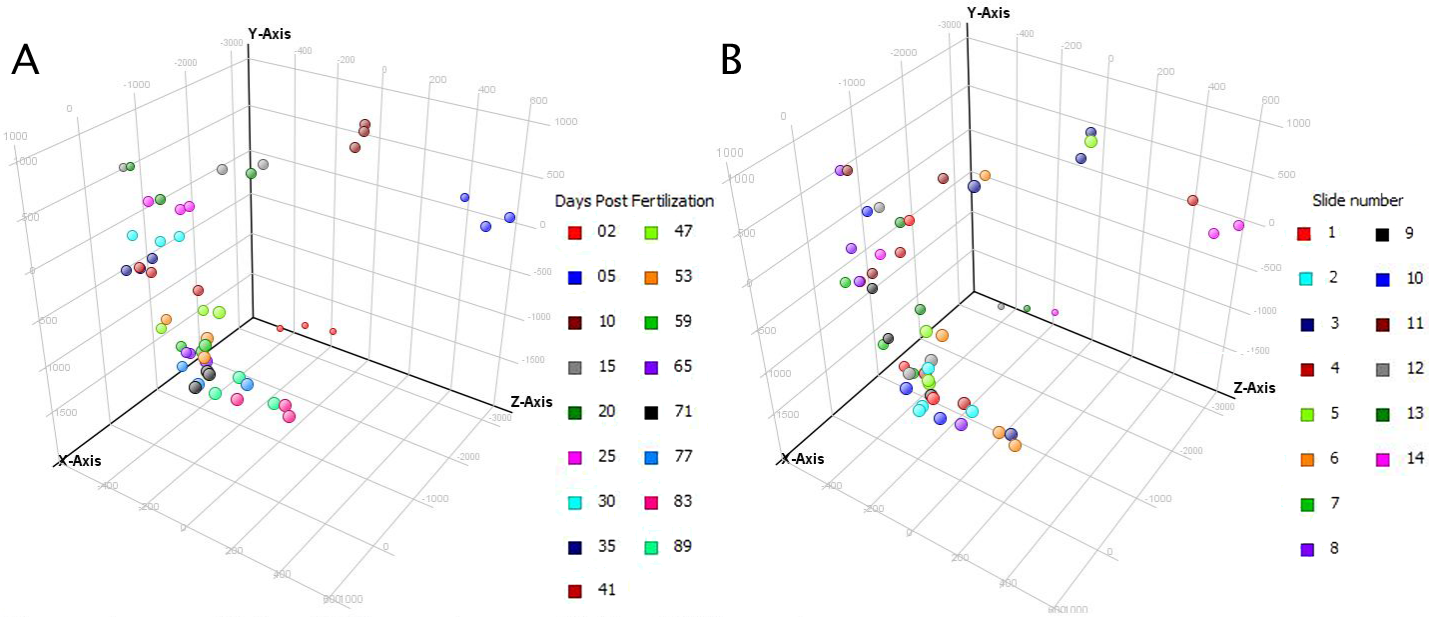
**3D-PCA plots showing variation based on experimental and technical variables**. Three-dimensional principle component analysis plots with each axis explaining a certain percentage of the variation among all samples. X-axis: 51.6% Y-axis: 21.9% Z-axis: 11.7% A) Samples colored according to days post fertilization B) Samples colored according to slide number.

This study complies with the MIAME standards [18]. Scanned arrays were quantified using Imagene 8.0 (Biodiscovery, El Segundo, CA) and processed with in-house scripts for input to GeneSpring GX 11.0 (Agilent). Data were imported into GeneSpring under the following conditions: raw data were converted to a threshold value of 1.0, data were log-transformed, a Lowess normalization was performed, and a baseline transformation to the median of all samples was performed. As a quality control measure, 3D-PCA graphs were examined with respect to the experimental variable, namely days post fertilization (Figure 2a), and technical variables such as slide number (Figure 2b), with four randomized samples per slide. Clustering according to days post fertilization was evident, with earlier days (2, 5, and 10) being further removed from the rest of the samples. In contrast, no real clustering was observable based on slide number, and this trend continued for other technical variables such as labelling day (data not shown). After data import, entities flagged for various reasons (e.g. poor spot morphology) by Imagene were filtered out, along with entities possessing raw signal values lower than 500.

As an initial exploratory analysis, conditions (dpf) were compared to each other using a sliding window approach. Using a t-test assuming unequal variance (Welch) (p ≤ 0.01, fold change ≥ 2.0) without multiple test correction (MTC), each time point was compared to the next time point in the series. This allowed a cursory examination of the genes that were differentially regulated as development progressed. Numbers of significantly differentially expressed entities are listed in Table 2 and lists of annotated entities are provided as supplementary tables (additional file 1, additional file 2, additional file 3, additional file 4, additional file 5, additional file 6, additional file 7, additional file 8, additional file 9, additional file 10, additional file 11, additional file 12, additional file 13, additional file 14, additional file 15, additional file 16). With a sample size of three individuals per condition, it is possible that discovery of more subtly changing differential regulation is limited and this may also cause inclusion of some false positives. Nonetheless, it is evident that high numbers of genes are being differentially regulated, especially in the first few weeks of development. In fact, it appears that the vast majority of transcriptional changes occur in the first 10 days of development.

**Table 2:** Numbers of differentially regulated entities across timeline

Comparison (dpf)	Number of differentially regulated entities
2 vs. 5	3329

5 vs. 10	2325

10 vs. 15	804

15 vs. 20	102

20 vs. 25	59

25 vs. 30	62

30 vs. 35	127

35 vs. 41	53

41 vs. 47	47

47 vs. 53	51

53 vs. 59	38

59 vs. 65	54

65 vs. 71	22

71 vs. 77	12

77 vs. 83	68

83 vs. 89	84

A Student's t-test assuming unequal variation (P ≤ 0.01, FC ≥ 2.0) without MTC was used to compare each sampled time point to the subsequent time point. Numbers of entities that were significantly differentially expressed between the compared time points are presented.

In order to investigate the biological and technical variation within and among conditions, we examined the variation among biological replicates for each entity (Table 3). All 23,854 entities that passed pre-filtering were used to determine the average standard deviation of replicates. The first condition (2 dpf) has the highest mean, indicating a possibly higher level of noise due to biological and technical variation; however the median is highest at 15 dpf, and in fact all days have comparable values. It appears that the data is not substantially noisier in the first days, therefore it is likely that the much higher number of differential transcripts in these comparisons is biologically accurate.

**Table 3:** Variation among replicate samples across timeline

Condition (dpf)	Mean	Median	Standard deviation
2	0.574	0.453	0.484

5	0.412	0.334	0.359

10	0.342	0.279	0.282

15	0.511	0.459	0.327

20	0.516	0.449	0.344

25	0.408	0.354	0.285

30	0.495	0.438	0.320

35	0.439	0.369	0.339

41	0.473	0.421	0.310

47	0.440	0.370	0.326

53	0.422	0.372	0.290

59	0.351	0.288	0.272

65	0.427	0.358	0.324

71	0.416	0.354	0.311

77	0.490	0.427	0.331

83	0.437	0.382	0.303

89	0.406	0.348	0.291

For each entity passing expression threshold and spot flag filters (total = 23,854), the standard deviation of the normalized, log-transformed values for the three replicates in a given condition was determined. For the calculated standard deviations within each condition, the mean deviation, median deviation, and standard deviation of the deviations were determined.

In terms of ontogenetically relevant probes on the 44K microarray, over 900 entities are currently annotated with the GO term "development". More specifically, approximately 620 and 180 entities are annotated with the terms "system development" and "embryo development", respectively. In this experiment, the majority of entities in each of these categories was expressed above our threshold of 500 in at least one condition. Some of the GO terms that are significantly enriched in the various comparisons include "blastocyst development" between 2 and 5 dpf, "brain development" between 5 and 10 dpf, "organ development" and "induction of apoptosis" between 10 and 15 dpf, and "erythrocyte development" between 15 and 20 dpf, to name just a few. Other researchers may perform fuller and more detailed analyses in accordance with their own questions and hypotheses.

## Use of dataset

Beyond this preliminary analysis, there is a wealth of information to be gained from these data and we have submitted all normalized and raw data to NCBI's Gene Expression Omnibus (GEO) [19] for others to examine. The data are accessible through GEO Series accession number GSE25938 http://www.ncbi.nlm.nih.gov/geo/query/acc.cgi?acc=GSE25938. This dataset encompasses variation among three individuals per condition and differences across 17 timepoints. It is evident that this microarray provides the ability to determine a detailed transcriptional basis of ontogeny and this experiment in particular contains a great deal of developmental information. In addition, these data could be used as a reference for perturbed or abnormal development in other studies, or for researchers to refer to when transcriptional patterns of specific genes are discovered in other young salmonids.

## Conclusion

Here we present a large and novel dataset that represents an invaluable source of information on the transcriptional changes present in developing salmon. We believe these data will be of interest to many researchers in several fields, including aquaculture, genomics, and developmental and evolutionary biology. Both as an examination of healthy development on its own and as a reference for future studies, this set of expression profiles will prove to be valuable to the scientific community.

## Competing interests

The authors declare that they have no competing interests.

## Authors' contributions

SGJ performed data processing and analysis and drafted the manuscript. DSS ran microarrays and performed initial data processing. KRVS performed animal sampling and RNA extractions. MY performed additional animal sampling and assisted with microarray reactions. FM designed the 44K microarray. BFK conceived of the study. All authors read and approved the final manuscript.

## Supplementary Material

Additional file 1 **02 vs. 05 annotated entities**. Spreadsheet of all annotated significantly differentially regulated entities between 2 and 5 dpf. Includes p-values, fold change, direction of regulation, and annotation information. Click here for file

Additional file 2 **05 vs. 10 annotated entities**. Spreadsheet of all annotated significantly differentially regulated entities between 5 and 10 dpf. Includes p-values, fold change, direction of regulation, and annotation information. Click here for file

Additional file 3 **10 vs. 15 annotated entities**. Spreadsheet of all annotated significantly differentially regulated entities between 10 and 15 dpf. Includes p-values, fold change, direction of regulation, and annotation information. Click here for file

Additional file 4 **15 vs. 20 annotated entities**. Spreadsheet of all annotated significantly differentially regulated entities between 15 and 20 dpf. Includes p-values, fold change, direction of regulation, and annotation information. Click here for file

Additional file 5 **20 vs. 25 annotated entities**. Spreadsheet of all annotated significantly differentially regulated entities between 20 and 25 dpf. Includes p-values, fold change, direction of regulation, and annotation information. Click here for file

Additional file 6 **25 vs. 30 annotated entities**. Spreadsheet of all annotated significantly differentially regulated entities between 25 and 30 dpf. Includes p-values, fold change, direction of regulation, and annotation information. Click here for file

Additional file 7 **30 vs. 35 annotated entities**. Spreadsheet of all annotated significantly differentially regulated entities between 30 and 35 dpf. Includes p-values, fold change, direction of regulation, and annotation information. Click here for file

Additional file 8 **35 vs. 41 annotated entities**. Spreadsheet of all annotated significantly differentially regulated entities between 35 and 41 dpf. Includes p-values, fold change, direction of regulation, and annotation information. Click here for file

Additional file 9 **41 vs. 47 annotated entities**. Spreadsheet of all annotated significantly differentially regulated entities between 41 and 47 dpf. Includes p-values, fold change, direction of regulation, and annotation information. Click here for file

Additional file 10 **47 vs. 53 annotated entities**. Spreadsheet of all annotated significantly differentially regulated entities between 47 and 53 dpf. Includes p-values, fold change, direction of regulation, and annotation information. Click here for file

Additional file 11 **53 vs. 59 annotated entities**. Spreadsheet of all annotated significantly differentially regulated entities between 53 and 59 dpf. Includes p-values, fold change, direction of regulation, and annotation information. Click here for file

Additional file 12 **59 vs. 65 annotated entities**. Spreadsheet of all annotated significantly differentially regulated entities between 59 and 65 dpf. Includes p-values, fold change, direction of regulation, and annotation information. Click here for file

Additional file 13 **65 vs. 71 annotated entities**. Spreadsheet of all annotated significantly differentially regulated entities between 65 and 71 dpf. Includes p-values, fold change, direction of regulation, and annotation information. Click here for file

Additional file 14 **71 vs. 77 annotated entities**. Spreadsheet of all annotated significantly differentially regulated entities between 71 and 77 dpf. Includes p-values, fold change, direction of regulation, and annotation information. Click here for file

Additional file 15 **77 vs. 83 annotated entities**. Spreadsheet of all annotated significantly differentially regulated entities between 77 and 83 dpf. Includes p-values, fold change, direction of regulation, and annotation information. Click here for file

Additional file 16 **83 vs. 89 annotated entities**. Spreadsheet of all annotated significantly differentially regulated entities between 83 and 89 dpf. Includes p-values, fold change, direction of regulation, and annotation information. Click here for file
